# HIV-1 Resistant CDK2-Knockdown Macrophage-Like Cells Generated from 293T Cell-Derived Human Induced Pluripotent Stem Cells 

**DOI:** 10.3390/biology1020175

**Published:** 2012-07-26

**Authors:** Marina Jerebtsova, Namita Kumari, Min Xu, Gustavo Brito Alvim de Melo, Xiaomei Niu, Kuan-Teh Jeang, Sergei Nekhai

**Affiliations:** 1 Center for Molecular Physiology Research, Children’s National Medical Center, Washington, DC 20010, USA; Email: mjerebts@childrensnational.org; 2 Department of Pediatrics, the George Washington University School of Medicine and Health Sciences, Washington, DC 20037, USA; 3 Center for Sickle Cell Disease, Department of Medicine, Howard University, Washington, DC 20059, USA; Email: Namita.kumari@howard.edu (N.K.); mxu2002@yahoo.com (M.X.); xniu@howard.edu (X.N.); 4 Molecular Virology Section, Laboratory of Molecular Microbiology, NIAID, National Institutes of Health, Bethesda, MD 20892, USA; Email: gbrito1998@yahoo.com (G.B.A.M.); KJEANG@niaid.nih.gov (K.-T.J.); 5 Department of Microbiology, Howard University, Washington, DC 20059, USA

**Keywords:** HIV-1 resistant macrophage-like cells, CDK2 knockdown, iPSC

## Abstract

A major challenge in studies of human diseases involving macrophages is low yield and heterogeneity of the primary cells and limited ability of these cells for transfections and genetic manipulations. To address this issue, we developed a simple and efficient three steps method for somatic 293T cells reprogramming into monocytes and macrophage-like cells. First, 293T cells were reprogrammed into induced pluripotent stem cells (iPSCs) through a transfection-mediated expression of two factors, Oct-4 and Sox2, resulting in a high yield of iPSC. Second, the obtained iPSC were differentiated into monocytes using IL-3 and M-CSF treatment. And third, monocytes were differentiated into macrophage-like cells in the presence of M-CSF. As an example, we developed HIV-1-resistant macrophage-like cells from 293T cells with knockdown of CDK2, a factor critical for HIV-1 transcription. Our study provides a proof-of-principle approach that can be used to study the role of host cell factors in HIV-1 infection of human macrophages.

## 1. Introduction

HIV-1 impacts patient health through a complex interplay with the host, but models to study the role of host genetics in this process are limited. Host tropism of HIV-1 is limited to human and chimpanzee that significantly reduces the number of available HIV-1 infection models. Human induced pluripotent stem cells (iPSCs) have the ability to produce host-specific differentiated cells, and thus have the potential to transform the study of infectious disease. To date, only a few iPSC models of infectious disease have been described [1,2]. Macrophages play a key role in HIV-1 infection. Although advances in mouse gene knockout technology have led to major contributions to the knowledge of murine macrophage development, human macrophages differ extensively from mouse cells and remain much less studied. Current methods for generating human primary macrophages vary in cell yield, purity, and activation status, often resulting in conflicting and difficult to interpret results [3,4]. The circulating monocytes are heterogeneous and vary in size, granularity, morphology and protein expression profile [4]. Several different monocyte subsets were characterized [5]. Moreover, the method of isolation influences the properties of differentiated macrophages and dendritic cells [6]. The primary human monocytes have a limited potential for proliferation *in vitro* [7] and are difficult to transfect [8]. Thus development of new approaches to produce a homogenous population of macrophages is very important. Furthermore, the phagocytic activity of these cells limits the ability for additional genetic manipulation. Genetically modified macrophages differentiated from human iPSCs can serve as a useful model for understanding the etiology of HIV-1 disease and facilitating the development of novel therapeutic interventions. 

Several combinations of transcription factors were successfully used for reprogramming human somatic cells into iPSC: Oct-4, Sox2, c-Myc and Klf4 (SY4 vectors cocktail) [9,10]; Oct-4, Sox2, Nanog and LIN28 (JT4 vectors cocktail) [11]; and Oct-4, Sox2, and SV40 T large antigen [12]. Major limitations of the current methods include low efficiencies of iPSCs production and differentiation, as well as non-efficient methods of genetic manipulation of iPSCs and primary human cells [13]. While *in vitro* reprogramming and subsequent differentiation can generate macrophages from any somatic cells, it is clear that many of the steps in this process need to be significantly improved. The choice of somatic cells for iPSCs generation is important due to the availability, maintenance costs, and epigenetis influence of the primary cells on the reprogramming and differentiation abilities [14]. While human primary fibroblasts and fibroblast cell lines are most often used for iPSC generation, we decided to use human embryonic kidney (293T) cells in our experiments. Previously, treatment of permeabilized 293T cells with mouse embryonic cell extracts led to the expression of Oct-4, Sox2, c-Myc and Klf4 genes [15] suggesting a possibility of 293T cells reprogramming into iPSC. Unlike primary fibroblasts, 293T cells can be effectively transfected and have unlimited proliferative resource thus offering a convenient model for optimization of reprogramming and differentiation protocols. Generation of iPSC from 293T cells offers the advantage of working with easy-to-produce knockout lines for differentiation into cells that are resistant for genetic manipulation.

In the present study, we have developed monocyte and macrophage-like cells from 293T cells that were reprogrammed into iPSC by transfection-mediated expression of Oct-4 and Sox2. Pluripotent clones were then differentiated into monocytes using a combination of interleukins IL-3/M-CSF, and the monocytes were differentiated into macrophage-like cells by incubation with M-CSF. To validate this method for production of genetically modify macrophages, we generated macrophage-like cells from 293T cells with CDK2 knockdown (KD). Our previous studies implicated CDK2 in the regulation of HIV-1 transcription [16,17]. Inhibition of CDK2 activity by siRNA [18], iron chelators [19,20], and roscovitin [21] inhibited HIV-1 replication, and prevented CDK2 association with HIV-1 promoter. Thus, CDK2 is an attractive target for anti-HIV-1 drugs development. In the present study, CDK2 knockdown macrophage-like cells were challenged with pseudotyped HIV-1 Luc virus, and demonstrated resistance for HIV-1 infection. Our study provides a proof-of-principle approach that can be used to study the role of host cell factors in HIV-1 infection of macrophages.

## 2. Results and Discussion

### 2.1. Results

#### 2.1.1. Stable CDK2 Knockdown in 293T Cells Inhibits HIV-1 Transcription

**Figure 1:**
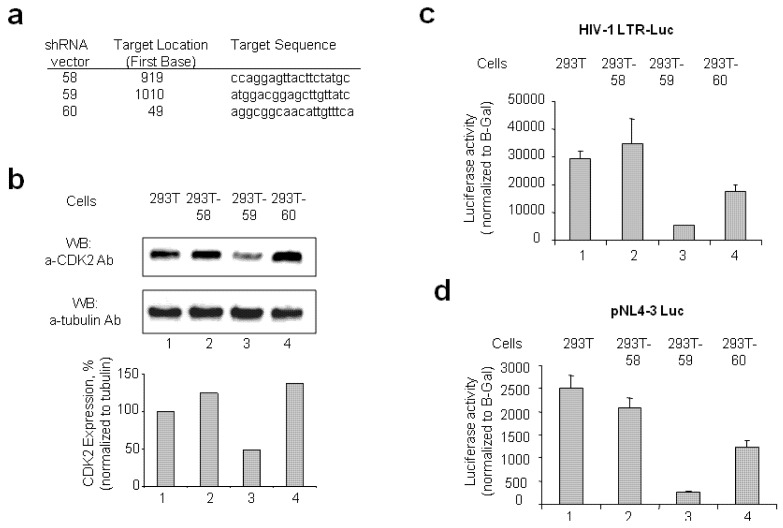
Expression of CDK2-targeted shRNA inhibits HIV-1 transcription.(**a**) Sequences in CDK2 targeted by shRNA-expressing vectors 58, 59 and 60. The sequences targeted by the indicated shRNA-expressing vectors and the target position in CDK2 gene sequence are shown. (**b**) Expression of CDK2 is reduced in 293T-59 cells. 293T cells were transfected with vectors expressing shRNA vectors 58 (lane 2), 59 (lane 3) and 60 (lane 4). Stables clones were selected with addition of puromycin. The selected cells were lysed in SDS-loading buffer and analyzed for the expression of CDK2 and α-tubulin as loading control. Lane 1, untreated 293T cells. Quantification of the western blot is shown as bar graph. (**c**) HIV-1 transcription is reduced in 293-59 cells. Control 293T cells, and cell lines stably expressing shRNA vectors 58 (293T-58), 59 (293T-59) and 60 (293T-60) were transfected with HIV-1 LTR-luciferase reporter and Tat-expressing vectors and also co-transfected with CMV-*Lac Z* vector for normalization purposes. Transfected cells were lysed at 48 hrs posttransfection the lyzates were assayed for the luciferase activity. The β-galactosidase activity was also measured and used for normalization. 293T-59 cells showed the greatest reduction of normalized luciferase activity. (**d**) HIV-1 genome transcription is reduced in 293-59 cells. The 293T, 293T-58, 293T-59 and 293T-60 cells were transfected with an HIV-1 genomic clone, pNL-4-3 Luc, that expressed luciferase in place of *nef* and also co-transfected the cells with CMV-*Lac Z* vector for normalization purpose. At 3 days posttransfection, the cells were lysed and luciferase activity was measured. The β-galactosidase activity was also measured and used for normalization. 293T-59 cells showed the greatest reduction of normalized luciferase activity.

We generated stable cell lines that expressed CDK2-targeting shRNAs using vectors that targeted three distinct sequences of CDK2 (Figure 1a). After transfection with each of the shRNA-expressing vectors and selection with puromycin, three cell lines were recovered expressing OS211958, OS211959 and OS211960 vectors that were designated as 293T-58, 293T-59 and 293T-60. Analysis of CDK2 expression in these cell lines indicated that only 293T-59 cells showed significant reduction of CDK2 expression (Figure 1b, lane 3). 

To analyze the effect of stable CDK2 knock-down on HIV-1 transcription, 293T-58, 293T-59 and 293T-60 cells were transiently transfected with HIV-1 LTR-luciferase reporter and Tat-expressing vectors and also co-transfected with CMV-*Lac Z* vector for normalization. Normalized luciferase activity was highly reduced in 293T-59 cells in comparison to 293T, 293T-58 and 293T-60 cells (Figure 1c, lane 3). To further analyze the effect of CDK2 knock down on HIV-1 transcription, we transfected 293T, 293T-58, 293T-59 and 293T-60 cells with an HIV-1 genomic clone, pNL-4-3 Luc, that expressed luciferase in place of *nef* and also co-transfected the cells with CMV-*Lac Z* vector for normalization. Again, 293T-59 cells showed the most significant reduction of normalized luciferase activity (Figure 1d, lane 3), suggesting that reduction of CDK2 expression suppressed HIV-1 transcription. We renamed 293T-59 clone as a 293T knockdown (293T-KD) and used it in further experiments.

#### 2.1.2. Reprogramming of 293T and CDK2 KD 293T Cells into iPSCs

**Figure 2:**
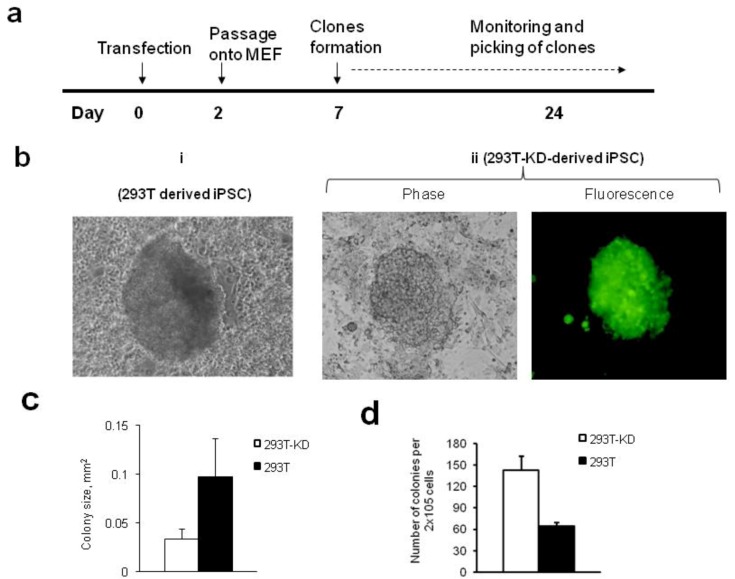
Generation of iPSC from 293T and 293T-KD cells. (**a**) A schematic diagram of the iPSC generation protocol. (**b**) iPSC representative images: (i) 293T derived iPSC colony; and (ii) 293T-KD derived iPSC colony, phase contract and GFP fluorescence images are shown. (**c**) Size of the colonies. Colonies were photographed and at least ten different colonies were measured. Results from three independent experiments are shown (**d**) Number of colonies generated from 293T and 293T-KD cells. About 2 × 10^5^ cells transfected with Oct-4 and Sox2 expression vectors were seeded per well and the iPSC colonies were counted. Results of three independent wells are shown.

We studied the feasibility of 293T cells reprogramming into iPSCs using a combination of Oct-4 and Sox2 expression vector. Previously, expression of SV40 large T antigen was shown to increase the efficiency of iPSCs production, and allowed the generation of iPSC colonies by expressing only two genes, Oct-4 and Sox2 [12]. Because 293T cells express SV40 large T antigen, we asked if the expression of Oct-4 and Sox2 can generate iPSCs from these cells. A schematic diagram of the iPSC generation protocol is shown in Figure 2a. 

We transfected 293T and 293T-KD cells with Oct-4 and Sox2/RFP expression plasmids, and the efficiency of transfection was monitored by the red fluorescence of RFP at 24 h after transfection (not shown). The 293T and 293T-KD cells were harvested 48 h after transfection, and plated on Mitomycin C treated MEF feeders and cultured with ES media. Six to eight days post transfection, ES-like colonies were formed. The colonies were small, dense and had irregular shapes with acute angles that are characteristic of ES-like colonies (Figure 2b, panels I and II). The colonies obtained from 293T-59 cells also showed GFP fluorescence (Figure 2b, panel II) suggesting that they retained the shRNA-expressing vector. No colonies were found in MEF cells co-cultured with non-transfected 293T or 293T-KD cells or in MEF cells cultured alone (not shown). The colonies generated from 293T-KD cells were smaller in size than 293T cells-derived colonies (0.034 ± 0.01 mm^2^ for 293T-KD and 0.098 ± 0.039 mm^2^ for 293T, Figure 2c). Interestingly, the number of colonies generated from 293T-KD cells was about two times higher than from 293T cells (143 ± 20 colonies from 293T-59 and 64 ± 5 colonies from 293T per 1 × 10^5^ transfected cells, Figure 2d).

**Figure 3:**
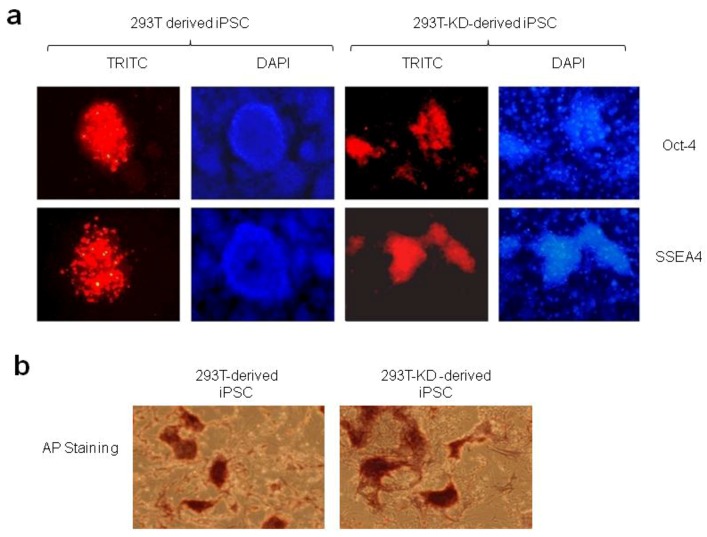
Characterization of iPSC colonies. (**a**) Immunofluorescence staining of ES markers. Oct-4 and SSEA4 markers staining in iPSC derived from 293T and 293T-KD cells, red fluorescence. DAPI staining is shown in blue fluorescence. Magnification-200×. (**b**) AP positive staining of 293T derived iPSC colonies. AP positive staining (red color) is shown for 293T and 293T-KD derived iPSC colonies.

To analyze the obtained iPSC, we examined the expression of undifferentiated cell markers (Oct-4 and SSEA4). Most colonies were positive for both Oct-4, and SSEA4 (Figure 3a). A few colonies were negative either for Oct-4 or SSEA4 (not shown). MEF, 293T and 293T-KD cells did not express SSEA4 and Oct-4 (not shown). We also did not observe RFP expression, suggesting that transient expression of Sox2 subsided during iPSC colonies formation. The colonies generated from 293T and 293T-KD cells expressed abundantly alkaline phosphatase (AP) (Figure 3b).

**Figure 4:**
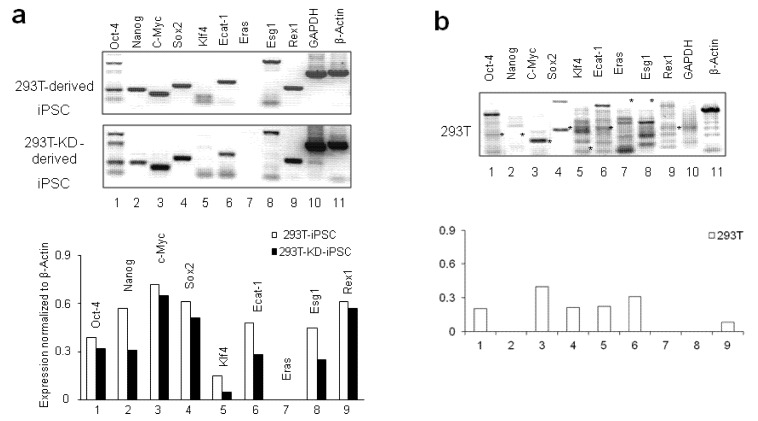
Expression of ES-specific genes by iPSC analyzed by RT- PCR. Expression of ES markers in iPSC derived from 293T and 293T-KD cells (**a**) and in parental 293T cells (**b**). Lane 1, Oct -4; lane 2, Nanog; lane 3, c-Myc; lane 4, Sox 2; lane 5, Klf4; lane 6, Ecat-1; lane 7, Eras; lane 8, Esg 1; lane 9, Rex 1; Lane 10, control GAPDH; lane 11, control β-Actin. Asterisks show position of the expected PCR products (panel b). Quantification of the results is shown.

We further characterized the iPSCs colonies by analyzing the expression of embryonic genes including Oct-4, Nanog, c-Myc, Sox2, Klf4, ES cell-associated transcript 1 (Ecat1) [22], Embryonic stem cell-expressed Ras (ERas) [23]; ES-specific gene-1 (Esg1) [24]; and expression gene 1 (Rex1) [25]. These embryonic genes were shown to be expressed in human ES and iPSCs with the exception of Eras that is expressed in mouse embryonic stem, but not in human embryonic stem cells [23]. Both 293T and 293T-KD cells-derived iPSCs expressed Oct-4, Nanog, c-Myc, Sox2, Klf4, Ecat-1, Esg-1 and Rex-1 (Figure 4a, lanes 1–6 and 8,9). No expression of Eras was observed as expected (Figure 3c and 3d, lane 7). The levels of Nanog, Ecat-1 and Esg-1 were lower in 293T-KD derived iPSCs than in 293T-derived iPSC (Figure 3d). Both iPSC expressed low levels of Klf4 (Figure 3d). We also analyzed the expression of these genes in parental 293T cells that expressed low levels of Oct-4, c-myc, Sox-2, Klf-4, and Rex1. Interestingly, 293T cells did not express GAPDH. Thus, the generated iPSCs expressed embryonic genes and stem cell markers. 

**Figure 5:**
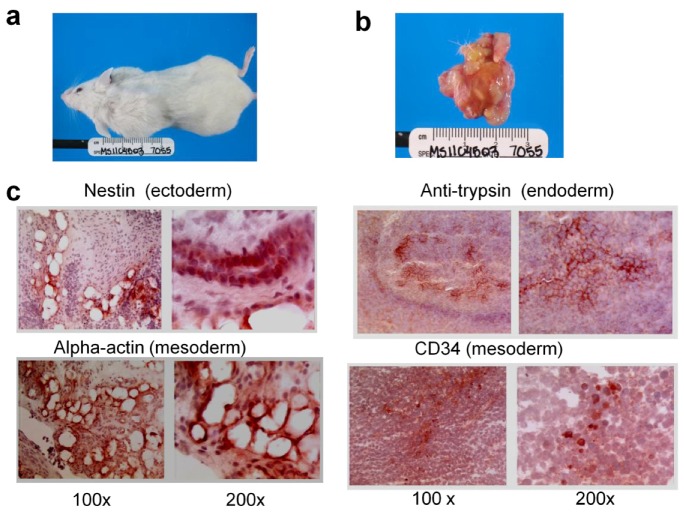
Characterization of tumors developed from iPSC. (**a**) Mouse with neck tumor. Representative picture is shown. (**b**) Isolated tumor. Representative picture is shown. (**c**) Characterization of tumor sections. Immunostaining for nestin , anti-trypsin, alpha-actin and CD34, red color. Hematoxyline (blue color) was used for counterstaining.

The 293T and 293T-KD iPSCs clones were differentiated into embryoid bodies (EB) and injected subcutaneously into the neck of NOD-SCID IL2Rg null mice (Figure 5a). We isolated the tumors and observed that they contained differentiated cells from all three germ layers (Figure 5b). The generation of nestin-positive neural precursor cells confirmed the presence ectodermal lineage (Figure 5c). The generation of alpha1-antitrypsin-positive hepatocytes-like cells confirmed the endodermal lineage (Figure 5c). The mesodermal lineage was confirmed by the generation of alpha- actin positive myocytes and multipotent HPCs positive for CD34 (Figure 5c). Thus, the transfection of 293T and CDK2 KD 293T-KD cells with only two transcription factors (Oct-4 and Sox2) sufficiently generated iPSCs that can differentiate into cells of three germ layers. 

#### 2.1.3. Differentiation of iPSCs into Monocytes

**Figure 6:**
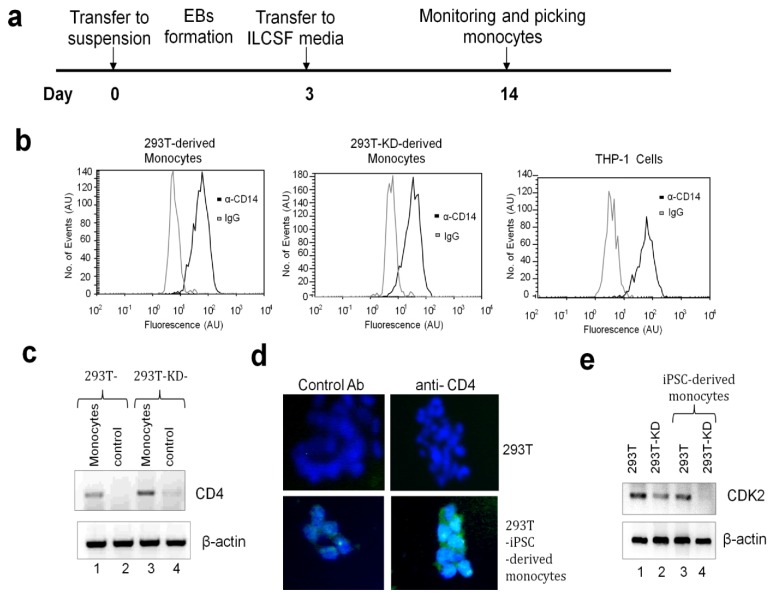
Differentiation of iPSC into monocytes. (**a**) A schematic diagram of the differentiation protocol (**b**) Analysis of CD14 expression in iPSC-derived monocytes. 293T iPSC-derived monocytes; 293T-KD iPSC–derived monocytes and control THP-1 cells were stained with APC-linked antibodies against CD14 and analyzed by FACS. Solid line: the cells stained with CD14 antibodies, shadow line: cells stained with non-specific IgG linked to APC. (**c**) Analysis of CD4 expression in iPSC-derived monocytes. RNA was isolated from 293T-derived monocytes (lane 1), 293T cells (lane 2), 293T-KD derived monocytes (lane 3), 293T-KD cells (lane 4). The RNA was reverse transcribed and amplified with primers specific for CD4 and β-actin as control. (**d**) Analysis of CD4 expression in iPSC-derived monocytes. Control 293T clls (upper panels) and the detached monocytes (lower panels) were fixed and immunostained with FITC-conjugated CD4 or non-specific mouse IgGs as indicated. Nuclei were counterstained with DAPI. (**e**) Analysis of CDK2 expression in iPSC-derived monocytes. RNA was isolated from 293T cells (lane 1), 293T-KD cells (lane 2), 293T-derived monocytes (lane 3) and 293T-KD derived monocytes (lane 4). The RNA was reverse transcribed and amplified with primers specific for CDK2 and β-actin as control.

For the differentiation of iPSC into monocytes, we adapted a protocol previously used for generation of homogeneous monocytes and macrophages from human embryonic stem cells [26]. A schematic diagram of the differentiation protocol is shown on Figure 6a. The iPSCs were transferred to ES media and allowed to form EB. Then the EB were cultured in the presence of IL-3 and M-CSF (ILCSF media). For 293T-KD-derived cells, the growth media was also supplemented with puromycin. After transfer of EB into ILCSF media, the EBs attached to the plate, and formed round monocyte-like cells after 10–14 days. The cells migrated from the attached EBs and detached (not shown). Cells produced from 293T-KD expressed high level of GFP (not shown). The detached cells were harvested from the media, and the expression of monocyte-specific markers was analyzed by immunofluorescence and flow cytometry. The cells were positive for monocyte marker CD14 (Figure 6b). The RT-PCR analysis also showed expression of CD4 in monocytes produced from iPSC (Figure 6c). Immunostaining also showed the presence of CD4 in monocytes produced from 293T-iPSC (Figure 6e). CDK2 expression was reduced in the parental 293T-KD cells and also in monocytes derived from 293T-KD iPSC as shown by RT- PCR analysis (Figure 6e). 

#### 2.1.4. Differentiation of iPSCs-derived Monocytes into Macrophage-like Cells

**Figure 7:**
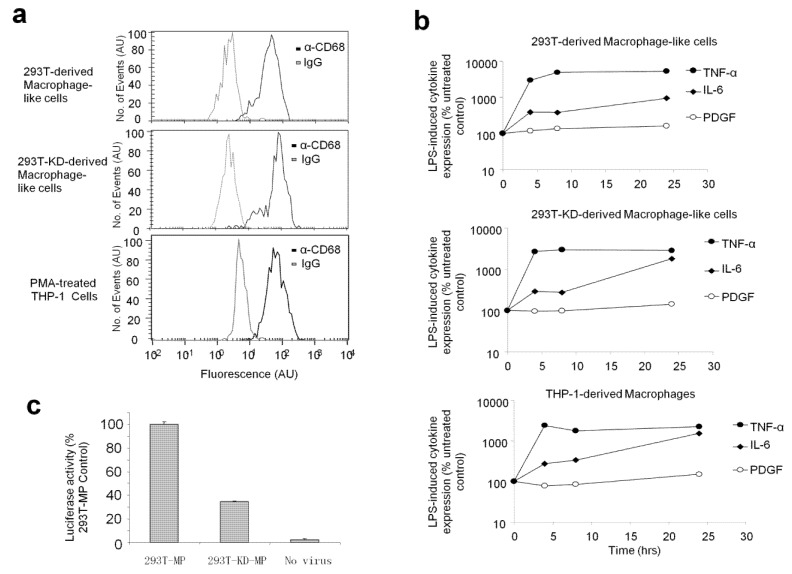
Characterization of iPSC-derived macrophages. (**a**) Analysis of CD68 expression in iPSC-derived macrophages. 293T iPSC-derived monocytes and 293T-5KD iPSC-derived monocytes were differentiated into macrophages by culturing with M-CSF. Control THP-1 cells were differentiated with PMA. The cells were stained with APC-linked antibodies against CD68 and analyzed by FACS. Solid line: the cells stained with antibodies; shadow line: cells stained with non-specific IgG. (**b**) Macrophages derived from 293T-iPSCs and 293T-KD-iPSCs produce cytokines in response to LPS stimulation. 293T and 293T-KD derived monocytes were differentiated into macrophages, and treated with LPS. Supernatant samples were collected prior to the LPS treatment and at 4, 8, and 24 hours of stimulation. Cytokines were analyzed by 10-plex Bio-Rad Multi-Plex assay. Induction of TNF-α and IL-6 and non-responsive PEDF are shown as percent of the initial concentration. (**c**) HIV-1 replication is reduced in macrophages differentiated from 293T-KD iPSC. Monocytes were differentiated into macrophages, infected with VSVG-HIV-1-Luc and cultured for 3 days. The cells were lysed and luciferase activity was measured. The activity was normalized to the cell number and presented as percent of activity in 293T-MP.

The monocytes were then differentiated into macrophage-like cells by culturing for 6 days with100 ng/mL M-CSF. The cells were evaluated by morphology and the expression of CD68, a marker expressed by the majority of human tissue macrophages [27]. Indeed, most cells differentiated into homogenous adherent macrophage-like population that express CD68 (Figure 7a). To test the cytokine profile of the macrophage-like cells, the cells were stimulated with LPS, and cytokines were measured using Bio-Plex array system (Bio-Rad). We found that the iPSC-derived macrophage-like cells secreted high levels of tumor necrosis factor-α (TNF-α) and IL-6, similar to the control macrophages differentiated from THP-1 cells (Figure 7b). No changes in the expression of platelet-derived growth factor (PDGF) (Figure 7b) and IL-8, IL-10, bFGF, IFNγ, MCP, RANTES and VEGF (not shown) were observed. Next, the iPSC-derived macrophage-like cells were infected with VSVG- pseudotyped HIV-1 Luc virus (Figure 7c). The macrophage-like cells derived from 293T CDK2 KD iPSC had reduced level of single cycle HIV-1 replication in comparison to the macrophage-like cells derived from 293T iPSC (Figure 7c).

### 2.2. Discussion

In this paper we have developed a platform for rapid, highly efficient and reproducible production of functional macrophages from 293T cells through the generation of iPSCs. This method provides a new model to study the role of host factors in the HIV-1 infection of macrophages and would be useful in the development of high-throughput approach for drug testing. In spite of the great progress in the development of iPSC and their differentiation into tissue-specific cells, further optimization is required before they become common laboratory methods. Recently, several iPSCs-based models of infectious disease have been described, including production of macrophages from human embryonic cells (ESC) and iPSC [1,2,28]. These studies demonstrated the feasibility of the method for iPSCs differentiation into functional macrophages with a normal phenotypic profile. Furthermore, introduction of lentiviral vectors expressing CCR5-targeting shRNA and a human/rhesus chimeric TRIM5α into somatic cells prior to their conversion to iPSCs demonstrated a strong protection against HIV-1 [28]. Here we made an attempt to optimize and simplify iPSCs production and differentiation into macrophages. The majority of recent methods of iPSCs production are based on the retroviral or lentiviral vectors delivery of reprogramming factors. Both viruses stably integrate into the host genome leading to constant expression of transforming factors that increases the risk of genetic modifications and reduces iPSCs potential for hematopoietic differentiation. Indeed expression of reprogramming genes Oct-4, Sox-2 and KLF-4 impedes hematopoietic development and has to be terminated prior to the differentiation [13,29]. Unfortunately, the reprogramming efficiencies of integration-free methods for iPSCs generation from human fibroblasts with adenoviral vectors, Sendai virus, polycistronic minicircle vectors and self-replicating selectable episomes are significantly lower than with viral vectors [30]. Thus the choice of somatic cell type for reprogramming plays an important role in the development of efficient protocols for iPSC generation with non-integrated vectors following the differentiation into hematopoietic cells. 

Several specific characteristics of 293T cells make these cells attractive for non-viral reprogramming into iPSC. First, the efficiency of transfection in 293T cells is significantly higher than in other cultured cell lines because of their ability for endocytosis which is a pivotal function of renal epithelial cells. The higher efficiency of transfection increases the probability of co-transfection of one cell with several plasmids that is necessary for efficient iPSCs production. Second, 293T cells express SV40 large T antigen that allows plasmids containing the SV40 origin of replication replicating to high copy number [31]. Thus SV40 large T antigen can increase the expression level of transforming factors in 293T cells. Indeed we found that more than 80% of cells expressed high levels of pSox2-RFP 24 h after transfection (not shown). To achieve this level of expression through infection with retroviral or lentiviral vectors will require much longer time because of an additional step of genome integration before productive expression. Recently it was demonstrated that the presence of SV40 large T antigen increases the efficiency of iPSC production by 20–70 folds and reduces the number of required factors from four to two, Oct-4 and Sox2 [12]. Here we generated iPSC that expressed pluripotent markers and produced *in vivo* tumor containing cells from three germ layers by simple transfection of 293T cells with two plasmids pSox2-RFP and pOCT3/4. We did not use c-Myc, the important factor for iPSC generation, for reprogramming, but our results demonstrated the expression of endogenous c-Myc protein in 293T cells that can substitute for ectopic c-Myc expression. Rapid expression of high levels of transforming factors likely contributed to the fast and efficient formation of ES-like clones in our system. Indeed we collected clones 6–15 days after transfection compared to the usual 15–30 days required after the viral vector delivery [9]. 

293T cells are widely used for production of cell lines with stable overexpression or knockout of a particular gene that opens a possibility to generate iPSC from the existing and new cell lines with alteration of gene expression. Our previous studies implicated CDK2 in the regulation of HIV-1 transcription [16,17]. Here we used 293T cells to generate a stable CDK2 knockout line expressing shRNA, and demonstrated that CDK2 knockout inhibited HIV-1 transcription in 293T cells. To study if CDK2 knockout also inhibits HIV-1 transcription in macrophages we first generated iPSC from the 293T cells with stable CDK2 knockout. The role of CDK2 in the reprogramming of human somatic cells into iPSC was not well understood, and there was a possibility that CDK2 KD cells had less efficiency for iPSC reprogramming or hematopoietic differentiation. Previously, it has been shown that downregulation of CDK2 in hESC causes G1 arrest, loss of pluripotency, upregulation of cell cycle inhibitors p21 and p27 and differentiation toward extraembryonic lineages [32]. Unexpectedly, the reprogramming of CDK2 KD cells in our study produced a higher number of iPSC clones than parental 293T cells. The average size of these clones was almost three times smaller than for 293T derived iPSC. This difference can reflect a higher propensity of CDK2 KD cells for reprogramming but also reflects slower rate growth that might be associated with longer G1 phase. The iPSC clones developed from both CDK2 KO and 293T cell lines expressed embryonic cells markers and produced tumors in mouse. The iPSC-derived tumors from both cell lines contained CD34+ positive hematopoietic stem cells. Thus reprogrammed 293T cells spontaneously differentiated *in vivo* into the hematopoietic stem cells. Epigenetic memory of somatic cells influents on the ability of the iPSC to differentiate into particular linage [13]. It is interesting that iPSCs generated from bone marrow using lentiviral vector demonstrated a reduced ability for hematopoietic differentiation despite their epigenetic memory [13]. It was attributed to the expression of an elevated level of iPSC transforming factors from integrated virus. In our experiments reprogramming factors were expressed only for a short period of time, and the expression of pSox2-RFP was undetectable in iPSC clones after 10–15 days (not shown). The loss of Sox2 and Oct-4 expression in the iPSCs colonies could improve the ability for hematopoietic differentiation. 

Next we investigate the potential of iPSC derived from 293T and CDK2 KD cells to differentiate into cells of myeloid linage *in vitro*. It has been reported that human iPSC derived CD34 cells stem cells were likely to differentiate into endothelial but not hematopoietic cells in contrast to mouse iPSC derived CD34 cells [33]. Several methods have been developed for production of monocytes and macrophages from pluripotent ES cells [34,35,36,37]. Current methods of monocyte production from human iPSC cells are complicated. They require co-culture with other cells type, the use of complex cytokine cocktails, and additional purification steps [38]. Recently, a simple method of monocytes production from hES trough embryoid bodies (EB) formation and EB culture with two cytokines IL-3 and M-CSF was developed [26]. Comparing the methods that use formation of monocytes on OS9/S17 mouse stromal feeder cells, this method is rapid, requires less intensive cell culture and gives rise to the homologues monocytes population. We adapted this method for monocyte production from human iPSCs. In our experiments, the monocytes were generated in 16–32 days, with 6–15 days to form the iPSC, three days of EB formation and 7–14 days to obtain detached monocytes. The frequency of iPSC colonies formation from transfected 293T cells was about 10^−3^. The number of monocytes differentiated from one iPSC colony was 10^7^–10^8^ cells. There were no significant differences between monocytes developed from both cell lines in terms of expression of CD68 and ability to secrete cytokines. Macrophage-like cells produced from both 293T and CDK2 KD cells were functionally similar to the macrophages differentiated from THP cell line. However, the macrophages derived from CDK2 KD cells were restrictive to HIV-1. We used VSVG-pseudotyped HIV-1 virus to simplify viral preparation and infection protocols and to bypass the requirements of having CD4 and a co-receptor for the infection. The VSVG-pseudotyped HIV-1 is about 100-fold more infectious and does not require nef function [39], which, in our construct, was deleted and substituted with a reporter luciferase gene. We were also interested in the analysis of HIV-1 transcription, which is affected by CDK2 KD [18], and one round replication by VSVG-HIV-1 Luc allows more accurate monitoring of HIV-1 transcription as opposed to multiple rounds of infection by HIV-1. Further analysis is needed to fully characterize the 293T iPSC-derived macrophage-like cells and to develop similar cells from primary epithelial cells. 

Taken together, we have developed a simple method of rapid and efficient production of large scale human monocyte and macrophage-like cells *in vitro* from human 293T cells through iPSCs generation. This method in combination with the ability for simple manipulation of host gene expression would provide a platform for investigation of human macrophage biology, and their role in development of HIV-1 diseases. 

## 3. Experimental Section

### 3.1. Materials

293T and THP-1 cells were purchased from ATCC (Manassas, VA). Mouse embryonic fibroblasts (MEF) were purchased from Applied Stem Cell (Sunnyvale, CA, USA). Paraformaldehyde, β-mercaptoethanol, Triton X-100, Mitomycin C, bovine serum albumin and goat serum were from Sigma-Aldrich (St. Louis, MO, USA). IL-3, M-CSF and FGF-2 were from R&D Systems (Minneapolis, MN, USA). Primary antibodies were mouse anti–SSEA-4 and mouse anti-Oct3/4 from BD Biosciences (San Jose, CA, USA), mouse anti-nestin from Chemicon (Temecula, CA, USA), mouse anti-alpha-actin, mouse anti-alpha1-antitripsin and mouse anti-tubulin from Sigma-Aldrich, mouse anti-CD34 and mouse anti-CDK2 from Santa Cruz Biotechnology (Santa Cruz, CA, USA). Secondary antibodies were biotinylated goat anti-mouse from Invitrogen (Rockville, MD, USA), and TRITC-conjugated and peroxydase–conjugated anti-mouse from Sigma-Aldrich. CD14-FITC was purchases from Becton Dickinson (Franklin Lakes, NJ, USA). CD14-phycoerythrin (PE), CD68-PE, AEC-substrate chromogen kit and BCIP/NBT kit were from Invitrogen (Rockville, MD, USA). 

### 3.2. Plasmids

Sox3 expression vector (piPSC-hSOX2-aRFP) was from SBI System Biosciences (Mountain View, CA, USA). Oct-4 expression vector (pCMV-Sport6h POU5F1) was from Open Biosystems (Huntsville, AL, USA). The HIV-1 genomic vector, pNL4-3.Luc.R^-^E^-^ (Courtesy of Dr. Nathaniel Landau) and VSVG-expressing vector, gpHEF-VSVG (courtesy of Dr. Lung-Ji Chang) were obtained from the NIH AIDS Research and Reference Reagent Program, Division of AIDS, NIAID, NIH. The Tat expression plasmid was a gift from Dr. Ben Berkhout (University of Amsterdam). WT HIV-1 LTR (–105 to +77) followed by the *luciferase* reporter gene (HIV-1 LTR Luc) was kindly provided by Dr. Manuel López-Cabrera (Unidad de Biología Molecular, Madrid, Spain) [40]. 

### 3.3. Luciferase Assays

293T cells were transfected using Lipofectamin and Plus reagents (Invitrogen) with HIV-1 LTR-Luc [40] and also with CMV-LacZ and Tat-expressing vectors [18]. Alternatively, the cells were transfected with NL4-3 Luc and CMV-LacZ expression vectors. After 48 hours the cells were collected, washed in phosphate-buffered saline (PBS), and lysed with Steady Lite Luciferase buffer (Perkin-Elmer, Bridgeville, PA). The luminescence was analyzed on Luminoskan (Perkin-Elmer). A portion of the lysate was used to measure β-galactosidase activity with o-nitrophenyl-β-D-galactopyranoside as previously described [18]. Luciferase activity was normalized on the basis of the obtained β-galactosidase activity.

### 3.4. Stable CDK2-knock-down Cell Line

293T cells were maintained in DMEM media supplemented with 10% FBS and 50 mg/mL streptmycin/penicillin (all from Invitrogen, Rockville MD). They were transfected with HSH000225-1-LvH1 vectors expressing shRNA that targeted ^919^ccaggagttacttctatgc^937^(OS211958), ^1010^atggacggagcttgttatc^1028^ (OS211959) and ^49^aggcggcaacattgtttca^67^ (OS211960) sequences of CDK2 (GeneCopoeia, Rockville, MD, USA). Stable clones were selected with 10 µg/mL puromycin (Invitrogen, Rockville, MD, USA). Clone OS211959 (designated as 293T-59) showed significant decrease in CDK2 expression and was further used for our studies.

### 3.5. Conversion of 293T Cells into iPSC.

293T cells and MEF cells were maintained in DMEM media supplemented with 10% FBS and 50 µg/mL streptomycin/penicillin (Invitrogen). 293T-59 cells were maintained in the same media supplemented with 10 µg/mL puromycin (Invitrogen). Conditioned media was purchased fromApplied Stem Cell (Sunnyvale, CA, USA). MEF were treated with Mitomycin C for 2 h, plated in 6-well plates at the density of 2 × 10^6^ cells/well, and cultured for two days to form a monolayer. 293T and 293T-59 cells were co-transfected in 60 mm plates with 1.5 µg piPSC-hSOX2-aRFP plasmid and 2.4 µg pOct-4 expressing plasmid and plated on MEF monolayer at 48 h posttransfection at concentration 1 × 10^5^ cells/well and cultured in embryonic stem cell (ES) media (DMEM, 10% FBS, 50 µg/mL streptomycin/penicillin, 5 ng/mL FGF-2 and 0.1mM β-mercaptoethanol). Formation of ES- like colonies was monitored daily. 

### 3.6. Tumor Formation

The iPSC clones were removed from the MEF feeder cells by mechanical pipette microdissection and cultured in MEF media to form embryoid bodies. The embryoid bodies were collected, mixed with DMEM containing 25% matrigel at 100 µL volume and injected subcutaneously into the neck of NOD-SCID IL2Rg null mice. Three mice were injected with 293T and 293T-59 derived iPSC, each with 0.5 × 10^6 ^cells. Tumors were formed at 4–6 weeks and were processed for immunohistochemical staining and analysis. All animal work was conducted at the Division of Veterinary Resources, Office of Research Services, National Institutes of Health according to approved animal study protocol. 

### 3.7. Differentiation of iPSC into Monocytes

The iPSC clones were removed from the MEF feeder cells by mechanical pipette microdissection and maintained in suspension in ES media without MEFs for 3 days. During this time, embryoid bodies (EB) were formed. The EBs were collected and differentiated into monocytes by transferring into ILCSF media (DMEM, 10% FBS, 25 ng/mL IL-3, 50 ng/mL M-CSF, 2 mM L- glutamine, 0.1 mM β-mercaptoethanol, and 50 µg/mL streptomycin/penicillin). 50–60 EB were used in each 6 well plate containing 3 mL of media. Media was replaced every 5 to 7 days. After transfer into ILCSF medium, EBs clones became attached and formed rounded monocyte-like cells. These detaching monocytes were harvested from the supernatant. Viability of the monocytes determined by Trypan blue dye exclusion method was about 80%–85%. Expression of monocytes- specific markers was monitored by immunofluorescence (IF) and flow cytometry. 

### 3.8. Differentiation of Monocytes into Macrophage-like Cells

About 2 × 10^6 ^293T iPSC and 293T-59 iPSC derived monocytes were differentiated into macrophage-like cells by culturing in complete DMEM media supplemented with 100 ng/mL M-CSF in 6 well plates , each well containing about 3 × 10^5^ cells. Medium was changed every 3 to 4 days by removing half and replacing culture media with twice the final concentration of M-CSF. As control, THP-1 cells were differentiated into macrophages by treatment with 10 nM phorbol 12-myristate 13-acetate (PMA, Sigma) for 48 hours [41].

### 3.9. Immunofluorescence Staining

The iPSC colonies were characterized by immunostaining with iPSC-specific anti–SSEA-4 and anti-Oct-4 antibodies and also stained for alkaline phosphatase activity (AP) using BCIP/NBT kit. For fluorescent immunostaining, iPSC colonies were fixed with 4% paraformaldehyde for 10 min, permeabilized with 0.5% Triton X-100, and blocked with 5% bovine serum albumin for 15 min. Cells were incubated with primary antibodies for 1 h following with TRITC-conjugated secondary antibodies for 30 min. Fluorescent images were analyzed using the Olympus IX51 fluorescent microscope equipped with DP72 digital camera. For immunostaining of monocytes, the detached monocytes were collected and smeared on a slide. Slides were fixed with 4% paraformaldehyde, blocked with 5% BSA, and incubated with FITC- conjugated CD14, CD16, CD86, HLA-DR, CD4, and CD57 antibodies or non-specific mouse IgG (Becton Dickinson, Franklin Lakes, NJ, USA) for 30 min. Nuclei were counterstained with DAPI (Sigma).

### 3.10. Immunocytochemistry of Tumor Sections

For characterization of three germ layers in the iPSC induced tumors, the 1.5–2 cm tumors were collected and kept fresh-frozen. Tumors were cut into the 4 μm sections, fixed in 4% paraformaldehyde for 10 min, blocked with 10% goat serum and immunostained with mouse anti-alpha-actin (Sigma Clone 1A4, 1:200 dilution), mouse anti-nestin (Chemicon, 1:200 dilution), mouse anti alpha1- antitrypsin (Sigma, 1:200 dilution) and mouse anti CD34 (Santa Cruz, 1:200 dilution). Secondary antibodies were biotinylated goat anti-mouse (Invitrogen). Then sections were incubated with streptavidin- peroxidase and developed using either AEC staining kit. Hematoxylin was used for counterstaining of sections.

### 3.11. RT-PCR

Total RNA was extracted from about 1 × 10^6^ iPSC 293T and 293T-59 cells using Nucleospin RNAII (Clontech, Mountain View, CA), according to the manufacturer’s protocol. Total RNA was reverse transcribed and semi-quantitative PCR was carried out using Pluripotency check PCR kit from Clontech. For RT-PCR of CD4 and CDK2, total RNA was isolated from cells using Trizol reagent (Invitrogen, Rockville, MD) and 5 μg of total RNA was used for first strand cDNA synthesis using SuperScript II First –Stand Synthesis Super Mix (Invitrogen). PCR was performed in 25 μL of Platinum PCR Supermix (Invitrogen) with primers for CD4 ( F 5’- CTC CCG CTC CAC CTC ACC CTG -3’; R 5’- GTG GAC CAA CCT TGA TGT TGG-3’), CDK2 (F 5’- TTTGCTGAGATGGTGACTCG – 3’; R 5’- CTTCATCCAG GGGAGGTACA) and β- actin (F 5’-GCT CGT CGT CGA CAA CGG CTC-3’; R 5’-CAA ACA TGA TCT GGG TCA TCT TCT C-3’). 

### 3.12. Flow Cytometry

For flow cytometry detached monocytes were collected. Macrophages were detached by incubating in trypsin for 5 minutes followed by wash in cold PBS containing 5 mM ethylenediamine tetraacetic acid (Sigma-Aldrich). THP-1 cells were used as a positive control for CD14 and THP1 cells differentiated using PMA as control for CD68. Cells were washed twice in PBS and incubated on ice with PE-labeled antibodies for 30 minutes PBS containing 10 μg/mL goat IgG and 1% fetal bovine serum. PE-labeled CD14 (monocyte marker) and CD68 (macrophage marker) antibodies were used. Results were analyzed on Agilent 2100 Bioanalyzer (Agilent technologies, Santa Clara, CA, USA) and 2100 Expert software (Agilent) was used to analyze the data. Data are presented as histograms with antibody staining in black relative to isotype-matched control in gray.

### 3.13. Cytokine and Chemokine Secretion

Macrophage-like cells derived from 293T iPSC and 293T-59 iPSC were incubated with 1 μg/mL lipopolysaccharide (LPS). Macrophage-differentiated THP-1 cells (48 hours with 10 nM PMA) were used as control. Supernatants from the LPS-treated cells were collected at 4, 8, and 24 hours of incubation and kept frozen at –20 °C. Human tumor necrosis factor (TNF)-α, IL-6, IL-8, IL-10, basic fibroblast growth factor (bFGF), IFNɣ, MCP, PDGF, RANTES and VEGF were measured using a custom-made 10-plex cytokine assay from Bio-Rad Laboratories (Hercules, CA, USA). The assay was performed using the Bio-Plex suspension array system with the manufacturer’s instructions (Bio-Rad). The system allows simultaneous identification of cytokines in a 96-well flat plate. In brief, the appropriate cytokine standards and samples were added to a 96 well flat plate. The samples were incubated at room temperature for 30 minutes with antibodies chemically attached to fluorescent-labeled magnetic micro beads. After three washes on Bio-Plex ProII wash station, premixed detection antibodies were added to each well and incubated for 30 min. Following three washes, premixed streptavidin-phycoerythrin was added to each well and incubated for 10 minutes followed by three more washes. Then beads were re-suspended with 125 µL of assay buffer and the reaction mixture was quantified using the Bio-Plex protein array reader. Data were automatically processed and analyzed by Bio-Plex Manager Software 6.0 using the standard curve produced from recombinant cytokine standard.

### 3.14. Infection of Macrophage-like Cells with HIV-1

Pseudotyped HIV-1 virus expressing luciferase was produced in 293T cells co-transfected with pNL4-3 Luc and VSVG-expressing vector as described [19]. Macrophage-like cells derived from iPSC or macrophage-differentiated THP-1 cells were infected with pseudotyped VSVG-HIV-1 at approximately 1 ng of p24 per 5 × 10^6^ cells. After 48 hours, the cells were collected, washed in PBS, and lysed with Steady Lite Luciferase buffer. The samples were then read in a Luminoskan and analyzed for luciferase activity. The luciferase activity was normalized by the cell number. 

## 4. Conclusions

We demonstrated that 293T and 293T-derived CDK2 KD cells can be efficiently reprogrammed into iPSC cells and then successfully differentiated into monocytes and macrophage-like cells. More importantly, we demonstrated reduced expression of CDK2 in inhibited HIV-1 transcription in both parental 293T cells and iPSC-derived macrophage-like cells. Thus our study provides a proof-of-principle approach that can be used to study the role of host cell factors in HIV-1 transcription in macrophages.
